# Dual-Mode Graphene Field-Effect Transistor Biosensor with Isothermal Nucleic Acid Amplification

**DOI:** 10.3390/bios14020091

**Published:** 2024-02-08

**Authors:** Hyo Eun Kim, Ariadna Schuck, Hyeonseek Park, Doo Ryeon Chung, Minhee Kang, Yong-Sang Kim

**Affiliations:** 1 Department of Electrical and Computer Engineering, Sungkyunkwan University, Suwon 16419, Republic of Korea; hyoeun0707@naver.com (H.E.K.);; 2 Biomedical Engineering Research Center, Smart Healthcare Research Institute, Samsung Medical Center, Sungkyunkwan University School of Medicine, Seoul 06351, Republic of Korea; juventus369@naver.com; 3 Department of Medical Device Management and Research, Samsung Advanced Institute for Health Science & Technology, Sungkyunkwan University, Seoul 06351, Republic of Korea; 4 Center for Infection Prevention and Control, Samsung Medical Center, Seoul 06351, Republic of Korea; 5 Division of Infectious Diseases, Department of Internal Medicine, Samsung Medical Center, Sungkyunkwan University School of Medicine, Seoul 06351, Republic of Korea

**Keywords:** multi-array, loop-mediated isothermal amplification, SARS-CoV-2, graphene field-effect transistor, colorimetric detection

## Abstract

Despite a substantial increase in testing facilities during the pandemic, access remains a major obstacle, particularly in low-resource and remote areas. This constraint emphasizes the need for high-throughput potential point-of-care diagnostic tools in environments with limited resources. Loop-mediated isothermal amplification (LAMP) is a promising technique, but improvements in sensitivity are needed for accurate detection, especially in scenarios where the virus is present in low quantities. To achieve this objective, we present a highly sensitive detection approach of a dual-mode graphene-based field-effect transistor (G-FET) biosensor with LAMP. The G-FET biosensor, which has a transparent graphene microelectrode array on a glass substrate, detects LAMP products in less than 30 min using both observable color changes and Dirac point voltage measurements, even in samples with low viral concentrations. This dual-mode G-FET biosensor emerges as a potential alternative to conventional RT-PCR for severe acute respiratory syndrome-associated coronavirus (SARS-CoV)-2 detection or point-of-care testing, particularly in resource-constrained scenarios such as developing countries. Moreover, its capacity for colorimetric detection with the naked eye enhances its applicability in diverse settings.

## 1. Introduction

The Coronavirus disease 2019 (COVID-19) pandemic posed substantial challenges to global public health systems. As the virus spread rapidly across continents, precise and prompt diagnostic methods became essential for disease detection, resource management, and containment [1,2,3]. Early detection of COVID-19 infections during the incubation period is crucial because of the virus’s ability to transmit to asymptomatic individuals [2,4,5]. Following the pandemic, respiratory diseases have recently re-emerged in unpredictable patterns [6]. Because the symptoms of COVID-19 and other respiratory diseases overlap, clinical diagnosis becomes challenging, necessitating reliance on laboratory testing for confirmation of infection [7]. Despite a significant increase in capacity during the pandemic, access to testing has emerged as a major obstacle, especially in low-resource and remote areas [8,9]. Limited availability of testing kits, reagents, and laboratory capacity impedes mass testing efforts, leading to delays in identifying positive cases and potential underreporting. Researchers and healthcare professionals have thus explored innovative approaches, such as point-of-care testing, to expedite diagnoses and reach underserved populations [10,11,12,13].

Isothermal amplification techniques, such as recombinase polymerase amplification and loop-mediated isothermal amplification (LAMP) have demonstrated promising results, offering high sensitivity, specificity, and speed under isothermal conditions [14,15]. LAMP is a point-of-care diagnostic tool that has promise for high-throughput screening in resource-constrained situations [16]. Its distinctive advantages include a rapid turnaround time for results and the capacity for colorimetric detection at end points using pH-sensitive dyes. However, improvements in assay sensitivity are needed for more accurate and reliable detection, particularly when viral quantities are low. Moreover, LAMP requires a set of four specific primers that recognize six distinct sequences on the target DNA, limiting the simultaneous detection of multiple genes in a single reaction. Therefore, the combination of LAMP with biosensors maximizing multiplex capabilities has been extensively investigated [8,14,17]. For example, electrochemical sensors with multiple electrodes that measure the redox activity of electroactive molecules resulting from their interaction with LAMP products allow for highly sensitive detection. This approach involves immobilizing a probe onto a working electrode coupled with a redox reporter, and the electrochemical signals are then meticulously monitored using techniques that include linear-sweep [18], square-wave [19], and differential-pulse voltammetry [17,20]. Additionally, label-free electrical sensing using field-effect transistors (FETs) has been employed to detect pH changes during amplification reactions with various geometries and gating methods [21,22]. Among these biosensing schemes, the graphene FET (G-FET) is distinctive owing to its exceptional capability for ultrasensitive and low-noise detection, facilitating real-time measurements, even with extremely low quantities of an analyte.

In this study, we developed a G-FET biosensor that used LAMP to detect the presence of severe acute respiratory syndrome-associated coronavirus (SARS-CoV)-2. Composed of a multi-array transparent graphene electrode, the G-FET biosensor can detect LAMP products through both color changes and Dirac point voltage measurements within 30 min.

## 2. Materials and Methods

### 2.1. Materials and Reagents

Monolayer graphene obtained through chemical vapor deposition (CVD) was sourced from Graphenea (San Sebastian, Spain). Polydimethylsiloxane (PDMS) was purchased from Dow Corning (Midland, MI, USA). Several other chemicals, including AZ-1512, SU-8 2075, diethyl pyrocarbonate (DEPC), hexamethyldisilazane, and (1-methoxy-2-propyl) acetate (SU-8 developer), were acquired from MicroChem (Westborough, MA, USA). Additionally, anhydrous iron (III) chloride (FeCl_3_) powder and phosphate-buffered saline (PBS; pH 7.4) were obtained from Sigma-Aldrich (St. Louis, MO, USA). WarmStart Colorimetric LAMP 2× Master Mix and LAMP Fluorescent Dye were purchased from New England Biolabs Inc. (Ipswich, MA, USA), and the primers synthesized and purified by Bionics Inc. (Seoul, South Korea). The template for the LAMP assay, consisting of a plasmid containing sequences of the SARS-CoV-2 envelope (*E*) and RNA-dependent RNA polymerase (*RdRP*) genes, was sourced from GenScript (Piscataway, NJ, USA).

### 2.2. Fabrication of Graphene Field-Effect Transistor Devices

We fabricated a five-electrode FET system for multiplex analysis. The technique used to create three coplanar electrodes was adapted from a previous study [23], where a 5 × 3 cm^2^ bare glass substrate was used. Initially, the glass substrates were cleaned by ultrasonication for 20 min each in acetone and isopropyl alcohol (IPA). They were then dried in an oven at 75 °C for 10 min. Once residue free, the bare glass was coated with a positive photoresist (PR) and pre-baked for 10 min at 95 °C. The glass/PR layers were subsequently exposed to ultraviolet (UV) radiation using an MA-6 Karl-Suss mask aligner. After developing the exposed PR, a thin gold (Au) film was deposited onto the glass/patterned PR layer with a thermal evaporator system at a 6.6 × 10^−6^ Torr vacuum level (Evaporation System, SHE-6T-350D). The fabricated glass/patterned PR/Au thin film layer was then soaked in acetone and ultrasonicated until excess PR was removed.

Next, monolayer graphene grown on a copper (Cu) layer via CVD was transferred to a glass substrate containing patterned gold electrodes. PDMS (Sylgard 184) was spin-coated onto the Cu/graphene sheet, and the thin PDMS layer cured in a drying oven for 20 min at 75 °C, following which the film was cut to a 40 mm × 3 mm piece to cover the entire five-electrode array and floated on an FeCl_3_ solution (0.25 mg/mL) to etch the Cu. The transparent graphene/PDMS sheet was rinsed with deionized (DI) water and transferred to the source and drain electrodes, whereafter it was baked in an oven for 5 min at 75 °C. The PDMS thin film was removed from the graphene using an acetone bath for 1 h, cleaned with IPA and DI water, and dried under nitrogen.

PDMS chambers were designed to incubate the LAMP assay solution that would interact with the graphene surface. The base and curing agents of Sylgard 184 were degassed for 1 h at 25 °C. These degassed agents were then poured onto a Petri dish in a 10:1 *v*/*v* ratio (base:cure) and cured for 4 h at 75 °C. The resultant solidified silicone elastomer was peeled from the Petri dish, and 5 mm diameter holes were punched therein. This elastomer was cleaned with acetone, IPA, and DI water, followed by UV-ozone treatment for 30 min. It was then aligned and bonded to the substrate, with the punched holes forming chambers over the active graphene layer.

### 2.3. RNA Extraction of Clinical Samples

RNA extraction was conducted using MagMax^TM^ Viral/Pathogen Nucleic Acid Isolation Kit and the KingFisher^TM^ Flex Purification System by Thermo Fisher Scientific Inc. (Waltham, MA, USA). In accordance with the manufacturer’s instructions, 530 μL of a binding solution, 20 μL of magnetic beads, and 10 μL of Proteinase K were sequentially mixed into each 200 μL sample for the extraction step. Subsequently, for the purification step, 1000 μL of a wash buffer, 1000 μL of ethanol (80% *v*/*v*), 500 μL of ethanol (80% *v*/*v*), and 50 μL of elution solution were utilized.

### 2.4. Loop-Mediated Isothermal Amplification Assay

The *E* and *RdRP* genes of SARS-CoV-2 were selected as target templates. These target template DNA samples were diluted with DEPC-treated water to a concentration of 1.48 × 10^5^ copies/μL. For the LAMP assays, the reaction mix contained target DNA (1 μL), a gene primer mix (forward inner (FIP; 1.6 μM), backward inner (1.6 μM, BIP), forward outer (0.2 μM, F3), and backward outer (0.2 μM, B3)), WarmStart Colorimetric LAMP 2× Master Mix (12.5 μL), LAMP Fluorescent Dye (0.5 μL), and ddH_2_O (9 μL). This LAMP assay reaction mix was then injected into the PDMS chamber, and the surface was covered with mineral oil to prevent evaporation. The temperature for the LAMP reaction was consistently maintained at 65 °C.

#### Measurement and Characterization 

Optimization of the LAMP assay was conducted utilizing the QuantStudio™ 6 Flex Real-Time PCR System by Thermo Fisher Scientific Inc. (Waltham, MA, USA). The assay was visually monitored with the naked eye, whereas a semiconductor parameter analyzer (HP Agilent 4145 B) was employed to assess the current–voltage (I–V) characteristics. Baseline measurements were conducted using a PBS solution. For data and statistical analyses, the Origin 8.5 software was used.

## 3. Results and Discussion

### 3.1. Characterization of Transferred Graphene

**Figure 1:**
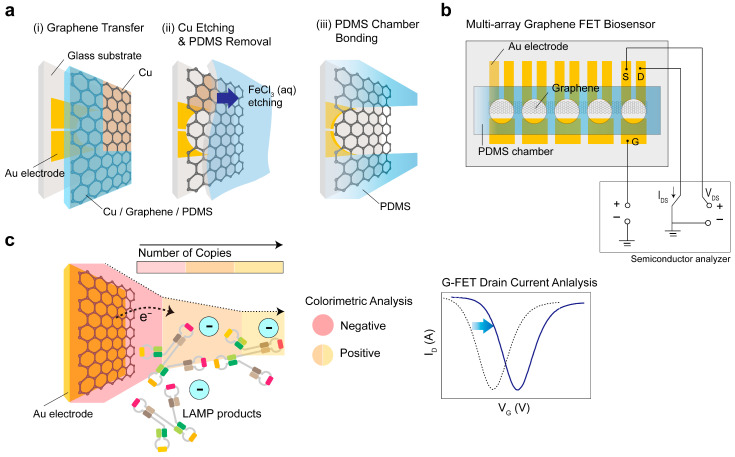
**Schematic diagram and functional concept of the graphene field-effect transistor (G-FET) biosensor.** (**a**) Chemical vapor deposition-grown graphene transfer accomplished using copper (Cu) as a sacrificial layer and polydimethylsiloxane (PDMS) as a supportive layer. (**b**) Design of the multi-electrode array featuring a graphene channel connecting the drain/source contacts, gate, and PDMS chamber. (**c**) Dual-mode monitoring illustrating the detection of amplicons through loop-mediated isothermal amplification (LAMP) via pH changes.

Figure 1 depicts the design and functional principles of the G-FET biosensor. The fabrication protocol utilized standard microelectromechanical system (MEMS) processes, using CVD-grown graphene. This material was transferred onto a multi-electrode array channel placed between drain/source contacts and the gate. The transfer process was carried out with Cu as a sacrificial layer and PDMS as a supporting layer (Figure 1a,b). To enable dual-mode monitoring, a PDMS chamber was firmly attached to a fully transparent graphene channel, allowing for efficient color change detection. Significant pH alterations caused by the LAMP products resulted in a discernible color shift in the sample: a positive reaction sample turned orange, whereas a negative reaction sample remained pink. Concurrently, the Dirac point voltage (V_Dirac_) showed a positive shift, which was attributed to a decrease in pH, indicating the p-doping effect induced by the negative charges of DNA on the graphene surface (Figure 1c) [24,25]. A comprehensive description of the fabrication process is provided in the Materials and Methods section. 

**Figure 2:**
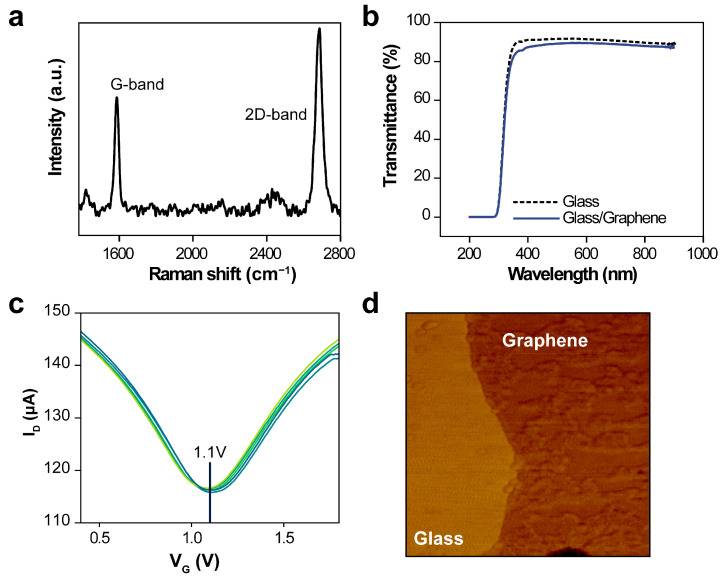
**Detailed analysis of the transferred graphene sheet**. (**a**) Raman spectra of the graphene sheet transferred to the multi-electrode array. (**b**) Ultraviolet–visible spectroscopy transmittance before and after transferring a single-layer graphene film to the glass substrate. (**c**) Transfer curves for the five-electrode array graphene field-effect transistor, with 1× phosphate buffer solution within the polydimethylsiloxane chambers. (**d**) Atomic force micrograph of the transferred graphene sheet.

The graphene, once transferred onto the multi-electrode array structure, was characterized by evaluating the intensity of the Raman spectra at a wavelength of 532 nm (Figure 2a). Peaks at 2683 cm^−1^ and 1586 cm^−1^, corresponding to the 2D and G bands, respectively, were identified. These observations confirm the successful deposition of a monolayer of graphene onto the electrode array [26,27].

Optical transparency of the device is essential for colorimetric measurements of sample solutions within a PDMS chamber. Figure 2b illustrates the transmittance of a single-layer graphene film transferred, over a wavelength range of 200–900 nm. The transmission spectra of both the reference glass substrates and graphene were normalized based on their respective transmittances in air. The average UV–Vis transmittances for the bare glass substrate and the combined glass/graphene layer were 90.67% and 88.52%, respectively, confirming the high transmittance of the graphene film.

The multi-electrode array structure was fabricated under consistent conditions to ensure uniformity of V_Dirac_. Such consistency and reliability in large-area graphene are crucial for integration into high-frequency measurement systems [27,28]. The transfer curve properties of G-FET sensors were assessed using a 1× PBS solution to evaluate stability across the five-electrode arrays (Figure 2c). The I–V characteristics of the G-FET devices were also recorded. Here, the gate voltage (V_G_) was varied from 0.4 V to 1.8 V in 0.01 V increments, and the drain current (I_D_) monitored, whereas the drain voltage (V_D_) was held constant at 0.4 V. When a V_G_ was applied, the ion concentration at the buffer solution–graphene interface increased. The G-FET displays ambipolar properties, and the charge-neutrality point on the I–V curve corresponds to V_Dirac_. Topologies of the transferred graphene sheets were further analyzed using atomic force microscopy (Figure 2d).

### 3.2. Optimization of the LAMP Assay

The LAMP assay was optimized through conventional benchtop PCR with eight sets of LAMP primers specifically designed to enhance detection of the SARS-CoV-2 *E* and *RdRP* genes. Detailed results obtained with the selected primer sets are provided in Table S1 of the Supplementary Information. Both colorimetric analysis and gel electrophoresis were employed to identify primers responsible for the most pronounced color change without causing non-specific reactions within a temperature range of 60–68.16 °C (Figure S1). The selected LAMP primers were associated with an optimal annealing temperature of 65 °C. Subsequent evaluation included the use of a serially diluted template, ranging from 1.48 × 10^8^ to 1.48 × 10^1^ copies per reaction for each target, to establish the limit of detection for the LAMP assay.

**Figure 3:**
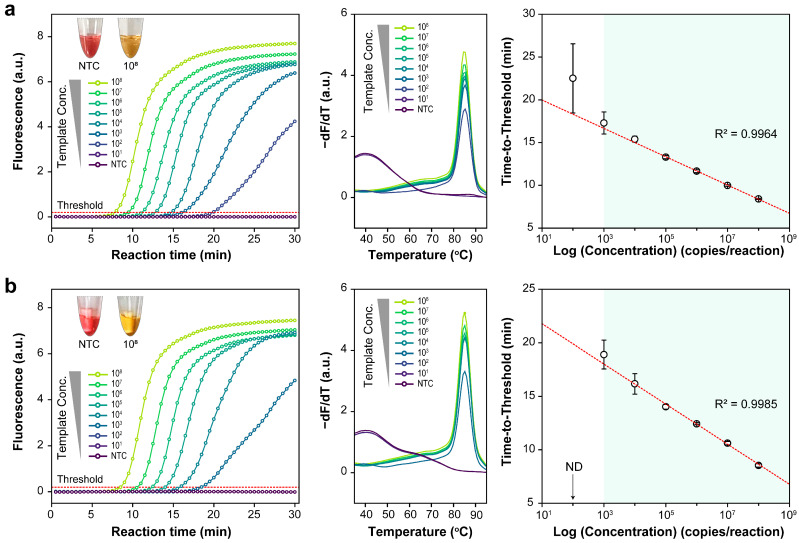
Loop-mediated isothermal amplification assay with ten-fold serially diluted templates ranging from 1.48 × 10^8^ to 1.48 × 10^1^ copies per reaction. Graphical representations of amplification plots, melting curves, and standard curves for the (**a**) *E* and (**b**) *RdRP* genes.

Figure 3 illustrates the amplification and melting curves of a series of targets, serially diluted 10×, using real-time fluorescence monitoring. The thresholds in the amplification curves were automatically determined by the software. Standard curves for the LAMP assay display plot of log concentration vs. time to threshold. Each target showed a strong linear connection (R^2^ > 0.99) over three repetitions., ranging from 1.48 × 10^8^ to 1.48 × 10^3^. These results suggest that the detection limit for the optimized LAMP reaction was approximately 1.48 × 10^3^.

### 3.3. Detection of SARS-CoV-2

**Figure 4:**
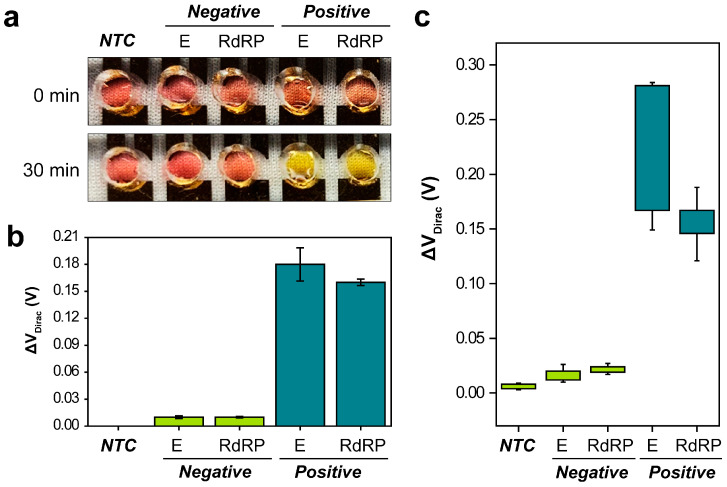
Dual-mode analysis of the loop-mediated isothermal amplification assay using the graphene field-effect transistor biosensor. (**a**) Colorimetric and (**b**) electrical analyses of the Envelope (*E*) and RNA-dependent RNA polymerase (*RdRP*) genes. (**c**) Dirac point voltage (ΔV_Dirac_) values for the clinical samples. NTC = non-template control.

After the LAMP reactions were carried out with specifically optimized primer sets that targeted the *E* and *RdRP* genes of SARS-CoV-2, chambers with a 50 mm diameter comprising PDMS were introduced with a non-template control (NTC), two negative assays, and two positive assays at a concentration of 10^5^ copies per reaction. The LAMP reaction was monitored both visually and using a semiconductor parameter analyzer. During the assay, the temperature of the G-FET biosensor was consistently maintained at 65 °C using a thermal hot chuck. After 30 min, the final amplified LAMP assay product was evaluated both colorimetrically and electrically through measuring the G-FET I_D_, as depicted in Figure 4a,b. For the colorimetric detection, an orange hue signified a positive reaction, whereas a neutral pink color denoted a negative reaction (Figure 4a). The I_D_ was assessed by sweeping V_G_ from −0.4 V to 1.6 V in increments of 0.01 V, with V_D_ held constant at 0.4 V (Figure 4b). Positive LAMP amplification led to a gradual reduction in the pH of the mixture. As a result of this pH decrease, the V_Dirac_ of the *E* and *RdRP* genes shifted positively by 0.18 V (standard deviation (SD), ±0.018 V; *n* = 3) and 0.16 V (SD, ±0.003 V; *n* = 3), respectively. In comparison, the V_Dirac_ of the negative reactions shifted only by 0.01 V (*E* gene: SD, ±0.0016 V; *n* = 3, and *RdRP* gene: SD, ±0.0007 V; *n* = 3).

For validation, 25 samples were evaluated and all samples containing the target genes were accurately identified as positive, and all controls and samples lacking the target genes were identified as negative. RT-PCR was conducted using *E* and *RdRP* genes, along with RNA extracted from clinical samples, to determine the cycle threshold (C*t*) values. The G-FET biosensor was used to measure NTC, 10 negative samples, and 10 positive samples (comprising five each of the *E* and *RdRP* genes) with a C*t* value < 30. The response of the G-FET biosensor for the LAMP measurement of clinical samples was characterized by the delta (Δ) voltage in the V_Dirac_. There was a noticeable shift of at least 0.149 V_Dirac_ and 0.121 V_Dirac_ in the positive *E* and *RdRP* genes, respectively, with C*t* values < 30. Conversely, changes of less than 0.027 V_Dirac_ were observed in the NTC and negative clinical samples. Comprehensive ΔV_Dirac_ values for each clinical sample can be found in Figure S2 of the Supplementary Information.

In summary, we introduce a dual-mode colorimetric and electrical G-FET biosensor designed for the detection of SARS-CoV-2 under isothermal conditions. Constructed using MEMS techniques for microelectrode array, our biosensor efficiently combines simplicity and effectiveness. Within a mere 30 min, this dual-mode biosensor successfully identified amplified target genes (SARS-CoV-2 *E* and *RdRP* genes) through both visual color changes and electrical monitoring of Dirac point voltage shifts. Our methodology not only streamlines the detection process but also significantly improves the economic feasibility and accessibility of the proposed method, particularly in resource-limited environments. In such settings, results can be observed qualitatively with the naked eye, eliminating the need for sophisticated equipment. Conversely, in well-resourced environments, the assay can be quantitatively measured using Dirac point voltage, leveraging the simplified G-FET biosensor technology. This dual-mode capability enhances the adaptability of the G-FET biosensor system to diverse environmental conditions.

## 4. Conclusions

In conclusion, our study introduces a versatile diagnostic method for detecting SARS-CoV-2 under isothermal conditions, featuring a dual-mode colorimetric and electrical G-FET biosensor. The integration of LAMP with G-FET electrical sensing enhances the biosensor’s capability to rapidly and accurately confirm COVID-19 infection without the requirement for expensive equipment. This approach provides an adaptable solution suitable for a variety of environmental settings.

## Data Availability

The original data and contributions presented in this study are included within the article or supplementary material. For further inquiries, please contact the corresponding author.

## Supplementary Materials

The following supporting information can be downloaded at: https://www.mdpi.com/article/10.3390/bios14020091/s1, Table S1: Information on the loop-mediated isothermal amplification primer sets used in the assay; Figure S1: Agarose electrophoresis results and naked-eye detection following the loop-mediated isothermal amplification assay; Figure S2: Dirac voltage shift values for each clinical sample.
