# Supplementary material for: Dual-Mode Graphene Field-Effect Transistor Biosensor with Isothermal Nucleic Acid Amplification
Source: Biosensors (Basel). 2024 Feb 8;14(2):91. doi: 10.3390/bios14020091 (PMC10886465; doi:10.3390/bios14020091)
Supplement: Supplementary file 1 [file biosensors-14-00091-s001.zip › biosensors-2821998-supplementary.pdf]

Supplementary Information

Dual-Mode Graphene Field-Effect Transistor Biosensor with Isothermal Nucleic Acid Amplification

Hyo Eun Kim [1], Ariadna Schuck [1], Hyeonseek Park [2,3], Doo Ryeon Chung [4,5], Minhee Kang [2,*], Yong-Sang Kim [1,*]

[1] Department of Electrical and Computer Engineering, Sungkyunkwan University, Suwon, 16419 Republic of Korea; hyoeun0707@naver.com (H.E.K.); arischuck@skku.edu (A.S.)
[2] Biomedical Engineering Research Center, Smart Healthcare Research Institute, Samsung Medical Center, Sungkyunkwan University School of Medicine, 06351, Seoul, Republic of Korea; juventus369@naver.com
[3] Department of Medical Device Management and Research, Samsung Advanced Institute for Health Science & Technology, Sungkyunkwan University, 06351, Seoul, Republic of Korea
[4] Center for Infection Prevention and Control, Samsung Medical Center, 06351, Seoul, Republic of Korea;
dr.chung@samsung.com
[5] Division of Infectious Diseases, Department of Internal Medicine, Samsung Medical Center, Sungkyunkwan University School of Medicine, 06351, Seoul, Republic of Korea

* Correspondence: minhee.kang@samsung.com (M.K.); yongsang@skku.edu (Y.-S.K.)

Target Region	Primer	Sequence	Amplicon (bp)
E	F3	GCTGATGAGTACGAACTTATGTAC	240
	B3	CAGAAGATCAGGAACTCTAGAAGA	
	FIP	AGGATGGCTAGTGTAACTAGCAAGA-GGAAGAGACAGGTACGTTAATAGTT	
	BIP	GCGCTTCGATTGTGTGCGT-CACGAGAGTAAACGTAAAAAGAAGG	
	LF	CGAAAGCAAGAAAAAGAAGTACGC	
	LB	CTGCTGCAATATTGTTAACGTGAG	
RdRP	F3	CTTGTTCTTGCTCGCAAACA	231
	B3	ACCATCAGTAGATAAAAGTGCA	
	FIP	CGCCACACATGACCATTTCACTCA-GTTGTAGCTTGTCACACCGTTTC	
	BIP	GGTGGAACCTCATCAGGAGATG-TAACATTGGCCGTGACAG	
	LF	ACTTGAGCACACTCATTAGCTAATC	
	LB	CCACAACTGCTTATGCTAATAGTGT	

Table S1: Information on the Loop-Mediated Isothermal Amplification Primer Sets Used in the Assay. Primers were designed using Primer Explorer V5 software (http://primerexplorer.jp/lampv5e/index.html).

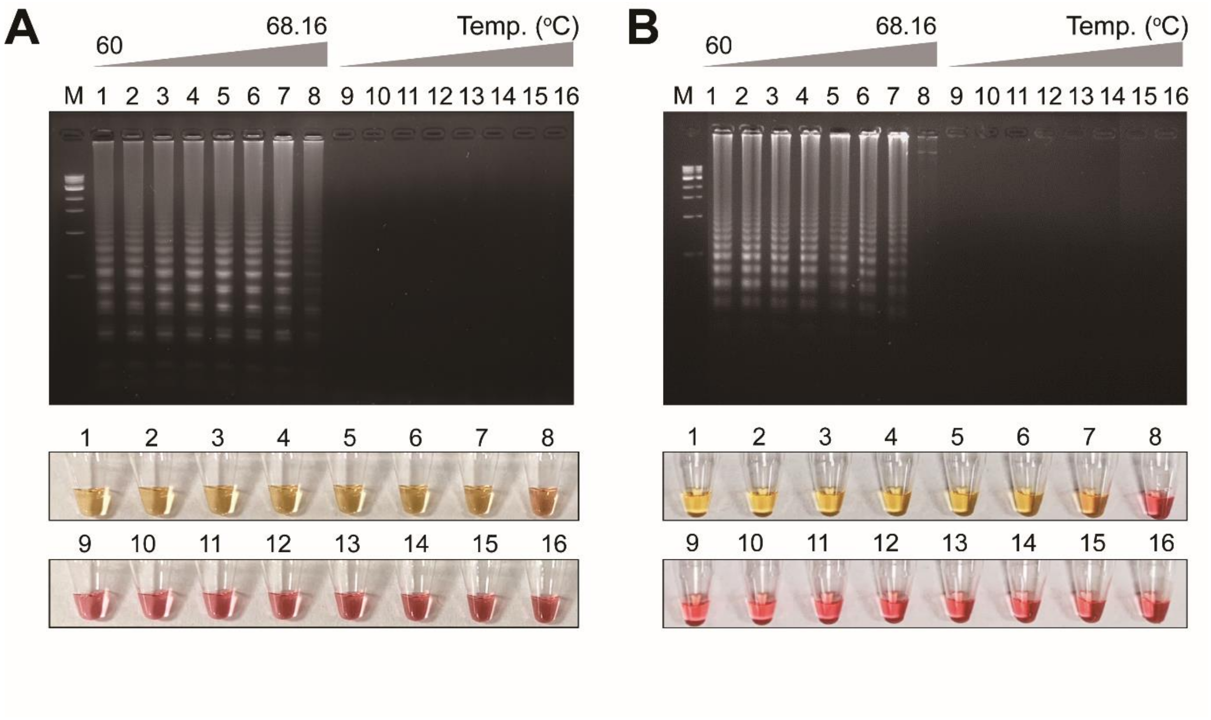

Figure S1: Agarose Electrophoresis Results and Naked-Eye Detection Following the Loop-Mediated Isothermal Amplification Assay. (A) E and (B) RdRP genes. Positive reactions display a distinct ladder of multiple bands in electrophoresis (lane M, 1 kb ladder; lanes 1-8, positive samples; lanes 9-16, non-template control; temperature gradient from 60-68.12 °C). In the colorimetric detection, orange signifies a positive reaction and neutral pink a negative reaction.

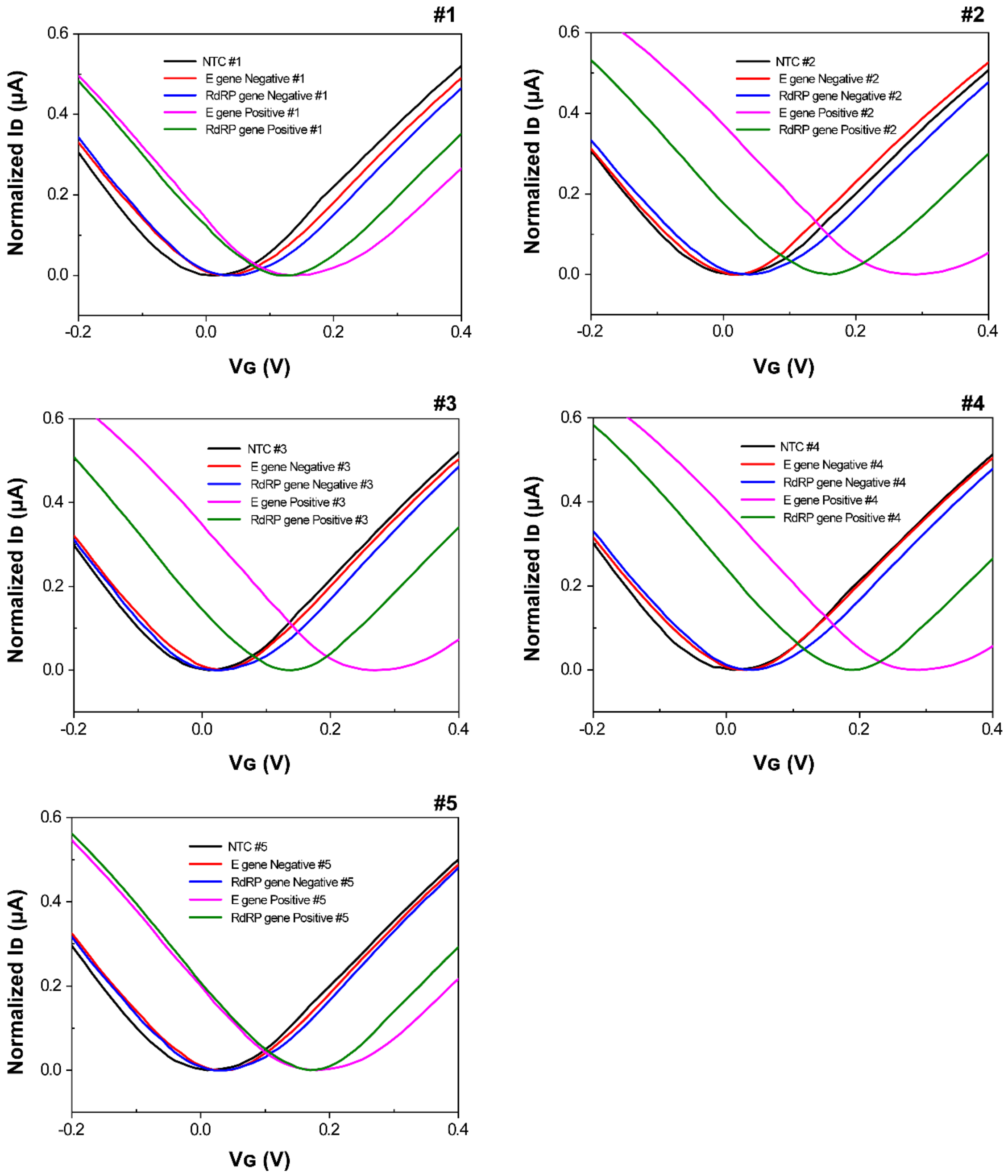

	ΔV_{Dirac} (V)				
	1	2	3	4	5
NTC	0.009	0.005	0.004	0.008	0.003
E gene (-)	0.020	0.010	0.016	0.012	0.026
RdRP gene (-)	0.019	0.024	0.017	0.027	0.020
E gene (+)	0.149	0.281	0.261	0.284	0.167
RdRP gene (+)	0.121	0.162	0.146	0.188	0.167

NTC, non-template control; ΔV_{Dirac}, shifts in the Dirac voltage.

Figure S2: Dirac voltage shift values for each clinical sample.
